# A case of arytenoid dislocation after ProSeal laryngeal mask airway insertion: A case report

**DOI:** 10.1016/j.ijscr.2024.110372

**Published:** 2024-09-30

**Authors:** Lingxi Xing, Yuyan Ding, Yihu Zhou, Lixiang Yu, Rong Gao, Lianbing Gu

**Affiliations:** a Department of Anesthesiology, The Affiliated Cancer Hospital of Nanjing Medical University, Jiangsu Cancer Hospital & Jiangsu Institute of Cancer Research, 210009 Nanjing, China.; b Department of Otorhinolaryngology Head and Neck, BenQ Medical Center, The Affiliated BenQ Hospital of Nanjing Medical University, 210019 Nanjing, China.

**Keywords:** Arytenoid dislocation, Arytenoid cartilage dislocation, Complications, Laryngeal mask airway

## Abstract

**Introduction and importance:**

Arytenoid dislocation, typically manifested as hoarseness and coughing when drinking, is a rare perioperative scenario, with an incidence rate of 0.009 %–0.097 % and endotracheal intubation under general anesthesia being the most common cause. However, arytenoid dislocation caused by a laryngeal mask airway (LMA) is extremely rare.

**Case description:**

Herein, a 53-year-old female patient was admitted for a “right breast lump” and scheduled for “unilateral mastectomy with ipsilateral axillary sentinel lymph node biopsy” under general anesthesia. During the surgery, the patient was noted to snore mildly, and rocuronium (15 mg) was immediately administered intravenously. The snoring ceased after adjusting the position of the LMA. Postoperatively, the patient was diagnosed with arytenoid dislocation by flexible nasal endoscopy after presenting with a sore throat accompanied by hoarseness and coughing when drinking. Thereafter, the patient underwent two cricoarytenoid joint reductions with a video laryngoscope under intravenous anesthesia, along with anti-inflammatory medication and voice therapy. The voice of the patient returned to normal after 1 month.

**Clinical discussion:**

Despite being a supraglottic airway device, the LMA can still cause arytenoid dislocation in clinical practice. Hence, anesthesiologists should analyze the potential causes and understand the diagnosis and treatment of arytenoid dislocation. Although closed reduction surgery typically requires two or three attempts, with a shorter disease duration leading to better outcomes, it can also aid in voice recovery for a longer disease course. In the presented case, the patient achieved a good prognosis after two closed reduction surgeries.

**Conclusion:**

Anesthesiologists should be vigilant for arytenoid dislocation in patients who present with persistent hoarseness and coughing while drinking after the insertion of the LMA, necessitating prompt treatment after diagnoses to achieve the best results.

## Highlights

• Arytenoid dislocation is a rare clinical complication.
• Arytenoid dislocation caused by a laryngeal mask airway is even more uncommon.
• Cricoarytenoid joint dislocation is prone to misdiagnosis or missed diagnosis.

## 1 Introduction

Arytenoid dislocation refers to the dislocation of the arytenoid cartilage from the cricoarytenoid joint and cricoid cartilage due to external force. Its clinical manifestations include hoarseness, stridor, coughing, and choking when drinking or swallowing, along with aphonia in some cases, making it a rare perioperative scenario. The first case of arytenoid dislocation was reported by Komorn et al. [1] in 1973. Reportedly, its incidence ranged from 0.009 % to 0.097 % [2,3]. Notably, Byron et al. [4] mentioned that arytenoid dislocation may be missed or misdiagnosed as recurrent laryngeal nerve injury, suggesting a higher actual incidence than reported. With the increasing number of surgeries involving general anesthesia and tracheal intubation, the incidence of arytenoid dislocation has been gradually increasing [5]. However, not many cases have been reported of arytenoid dislocation caused by laryngeal mask airway (LMA) insertion. In 1996, Rosrnberg et al. [6] first reported the incidence of arytenoid dislocation due to LMA. To date, only two case reports of LMA-mediated arytenoid dislocation have been submitted to the Pubmed and Web Of Science databases [7]. This article reports the diagnosis and treatment process of arytenoid dislocation following the insertion of an LMA in a patient undergoing breast surgery under general anesthesia. We have reported this case report in line with the SCARE criteria [8].

## 2 Case report

**Fig. 1:**
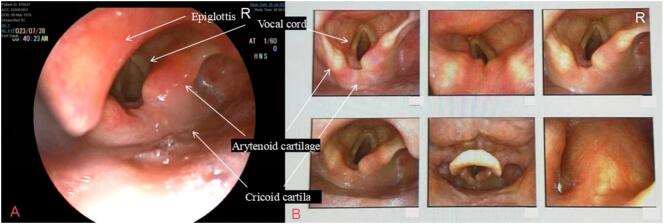
A: Flexible nasal endoscopy performed at the Affiliated Cancer Hospital of Nanjing Medical University. **B**: Flexible nasal endoscopy performed at the Affiliated BenQ Hospital of Nanjing Medical University. **Note**: Flexible nasal endoscopy revealed normal activity in the left arytenoid region, but poor activity in the right arytenoid region. The right vocal cord was fixed, and the vocal cord closure was poor. The mucosa of the bilateral vocal cords and arytenoid regions was normal.

**Fig. 2:**
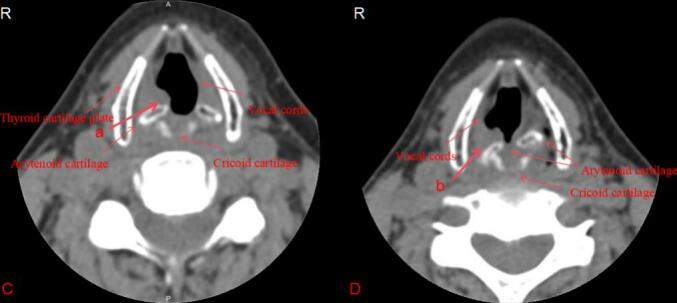
**C**: Breathing phase **D**: Phonation phase. **a**: The right vocal cord does not open completely, while the left vocal cord opens normally. **b**: The right vocal cord does not fully adduct, while the left vocal cord is at the midline. The glottis does not close completely.

**Fig. 3:**
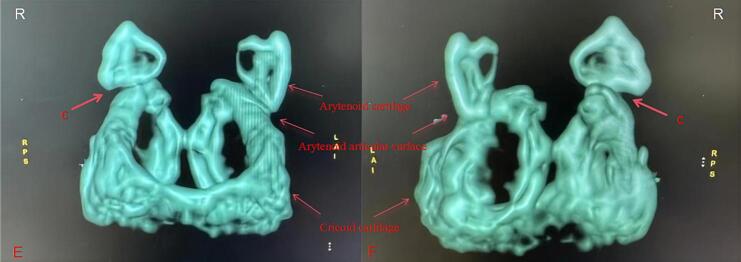
E: Anterior view of the cricoarytenoid joint **F**: Posterior view of the cricoarytenoid joint. **c**: VR shows a slight posterior displacement of the right arytenoid cartilage, exposing the anterior part of the joint surface. No significant abnormality was noted in the position of the left arytenoid cartilage.

**Fig. 4:**
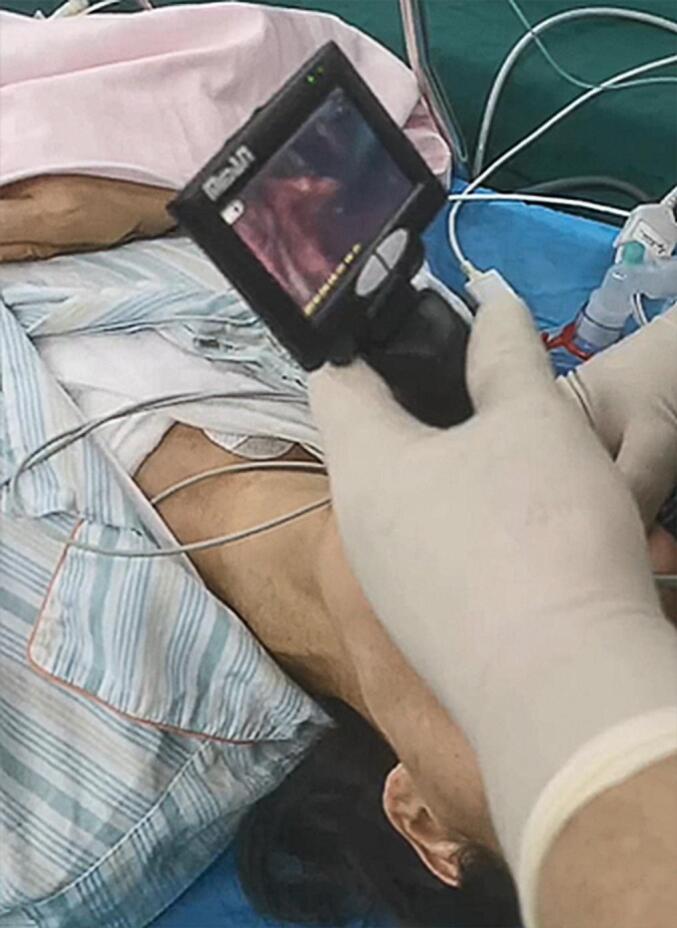
The patient underwent cricoarytenoid joint reduction with video laryngoscopy.

**Fig. 5:**
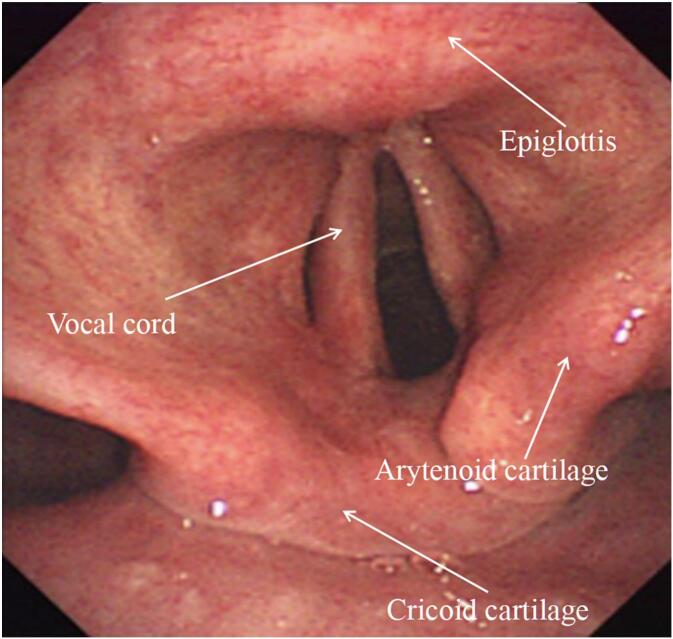
Flexible nasal endoscopy was performed after 7 days of treatment. **Note:** Flexible nasal endoscopy revealed that the bilateral arytenoid cartilages were positioned on the same plane, with the right arytenoid cartilage exhibiting a slight swelling.

A 53-year-old female patient (height: 163 cm, weight: 48 kg, body mass index [BMI]: 18.1) was admitted to the Affiliated Cancer Hospital of Nanjing Medical University on July 24, 2023, due to a “right breast lump.” The patient had no history of surgery or trauma, showed good physical activity tolerance, and had no symptoms of chest tightness, chest pain, cough, sputum, or difficulty in breathing. Following admission, comprehensive routine examinations were conducted which revealed normal preoperative blood routine and liver and kidney functions, along with a normal electrocardiogram (ECG). There were no contraindications for surgery, and the patient was classified as an American Society of Anesthesiologists (ASA) class II patient, with a Mallampati airway classification of grade II, presenting normal tracheal positioning and normal neck mobility. On July 26, 2023, the patient underwent “unilateral radical mastectomy with ipsilateral axillary sentinel lymph node biopsy” under general anesthesia. After entering the operating room, the blood pressure, oxygen saturation, heart rate, ECG, and anesthesia depth of the patient were monitored using SedLine, a multimodal brain function monitor. After establishing the intravenous access, the patient was pre‑oxygenated and administered with midazolam (1 mg), dexamethasone (5 mg), sufentanil (0.4 μg·kg^−1^), 1 % propofol (1 mg·kg^−1^), rocuronium (0.6 mg·kg^−1^), and hydrocortisone (5 mg). A size 3 ProSeal LMA was selected and successfully inserted in the first attempt, with an intra-cuff pressure of 35 cmH_2_O. Manual ventilation was performed without leakage, with good chest rise and fall and normal breath sounds, end-tidal partial carbon dioxide (PETCO_2_), and airway pressure waveforms. Anesthesia machine parameters were adjusted as follows: volume-controlled ventilation (VCV) mode was used; tidal volume, 8 ml·kg^−1^ (predicted body weight); positive end-expiratory pressure (PEEP), 5 cmH_2_O; fractional inspired oxygen, 0.5; inspiratory to expiratory ratio, 1:2; and respiratory rate adjusted to maintain PETCO_2_, 35–45 mmHg. During the surgery, the patient was continuously administered with propofol (0.04–0.06 mg·kg^−1^·min^−1^), remifentanil (0.2 μg·kg^−1^·min^−1^), rocuronium (0.01 mg·kg^−1^·min^−1^), and dexmedetomidine (16 μg·h^−1^) to maintain the Patient State Index between 25 and 50. A 3 M inflatable warming blanket was used intraoperatively to maintain the nasopharyngeal temperature above 36 °C. The vital signs of the patient remained stable throughout the surgery, which was successful and lasted 202 min, and no vasoactive drugs were administered. Notably, the patient was mildly snoring 30 min before the end of the surgery, and hence, was administered intravenous rocuronium (15 mg) and the LMA position was adjusted, which resulted in cessation of snoring. During the surgery, approximately 10 ml of blood loss occurred. At post-surgery 15 min, the patient regained full consciousness, exhibiting responsiveness and restored pharyngeal and swallowing reflexes. The tidal volume and time of ventilation returned to normal, and the LMA was smoothly removed. The next day (July 27, 2023), the patient complained of a sore throat accompanied by hoarseness and coughing when drinking water. Owing to the possibility of the cause being anesthesia-related factors, along with inflammation and edema, an anesthesiology consultation was requested for symptomatic management, and the temporary administration of dexamethasone was continued, with consideration for flexible nasal endoscopy, if necessary. On the post-operative 2nd day (July 28, 2023), the symptoms of sore throat, hoarseness, and coughing while drinking water did not improve. Thereafter, a flexible nasal endoscopy was performed after consulting with the otolaryngology department (Fig. 1A). The results of “poor movement of the right arytenoid and vocal cord, inadequate glottic closure” raised suspicion for arytenoid dislocation. After primary diagnosis, the patient was transferred to the Department of Otolaryngology Head and Neck Surgery at the Affiliated BenQ Hospital of Nanjing Medical University on the same day (July 28, 2023). A subsequent flexible nasal endoscopy revealed (Fig. 1B) “adequate mobility of the left arytenoid area, poor mobility of the right arytenoid area, fixed right vocal cord, and inadequate vocal cord closure.” The right arytenoid dislocation was confirmed after a computed tomography scan of the cricoarytenoid joint (Fig. 2) and volume rendering (Fig. 3). On the postoperative 5th day (July 31, 2023), cricoarytenoid joint reduction with video laryngoscope under intravenous anesthesia (the spontaneous breathing of the patient was maintained) was performed at the Affiliated BenQ Hospital of Nanjing Medical University (Fig. 4). Despite the smooth proceedings of the surgery, the patient continued to experience hoarseness and coughing while drinking water. Therefore, the patient underwent a follow-up examination on August 8, 2023, and the next day (August 9, 2023), another cricoarytenoid joint reduction with a video laryngoscope under general intravenous anesthesia was performed. Postoperatively, the vital signs of the patient remained stable, and improvement was observed in hoarseness compared with that observed before. There was still slight throat pain and discomfort while swallowing postoperatively; therefore, the patient was treated with nebulized hydrocortisone and voice therapy for 1 week. Another flexible nasal endoscopy (Fig. 5) showed that the structure and function of the right cricoarytenoid joint returned to normal. At the 1-month follow-up visit (September 8, 2023), the patient showed complete recovery, with no difficulty in phonation, hoarseness, or throat pain.

## 3 Discussion

Arytenoid dislocation can be caused by various factors in clinical practice, which are mainly classified into four different categories [9], namely patient, anesthesia-related, surgical, and invasive procedural factors. Patient factors include congenital malformation of the arytenoid joint, low BMI, and difficulty in glottic exposure during airway assessment. Anesthesia-related factors include endotracheal intubation and improper use of endotracheal tube cuffs and laryngoscopes. Surgical factors include prolonged surgery with endotracheal intubation under general anesthesia, multiple changes in patient positioning during the surgery, and improper surgical techniques involving the larynx. Invasive procedural factors include the improper placement and retention of gastric tubes and the use of trans-esophageal echocardiography probes.

The ProSeal LMA [10,11] is an upgraded version of the classic LMA and is widely used in clinical practice. It offers the advantages of aspiration and reflux prevention, a simpler insertion procedure, minimal patient discomfort, and higher tolerance, making it a relatively safe supraglottic airway device. When correctly inserted, the ProSeal LMA typically does not affect the arytenoid joints. However, because of the positioning of the distal end of the LMA cuff, inflation may cause slight anterior displacement of the arytenoid cartilage. Moreover, in the case of LMA displacement, significant displacement of the arytenoid cartilage may occur, potentially leading to arytenoid dislocation. Common LMA-associated causes of arytenoid dislocation include LMA-induced pressure on the arytenoid cartilage, rotation or compression of the LMA during insertion or post-insertion, excessive insertion of the laryngoscope blade during assistance in LMA placement, excessive inflation of the LMA cuff, and inadequate depth of anesthesia leading to swallowing activity or coughing.

The analysis of the arytenoid dislocation in the present case study indicated several potential causes and their combination effect possibly contributed to the incidence of arytenoid dislocation.

Regardless of the smooth insertion of the LMA under rapid induction of general anesthesia, blind insertion techniques for LMA placement cannot guarantee avoidance of contact with sensitive structures such as the epiglottis during insertion. The surgery lasted for 202 min, and slight snoring was noted 30 min before the end of the surgery. It may be because the level of muscle relaxation might have become shallower, leading to swallowing movements that caused the LMA to shift, possibly involving the cricoarytenoid joint. Prolonged surgery duration possibly prolonged the LMA-induced pressure on surrounding tissues, causing damage and increasing the likelihood of arytenoid dislocation. Additionally, the patient had a slender physique and a low BMI, which have been reported as independent risk factors for arytenoid dislocation [9,12]. Bene et al. [13] reported that using muscle relaxants during general anesthesia can relax the arytenoid joints, which inherently possess shallow joint surfaces, lax joint capsules, and easy mobility.

Closed reduction surgery (arytenoid cartilage manipulation) is the preferred treatment for arytenoid dislocation and serves as both diagnostic and therapeutic measures. However, the timing of the reduction remains controversial in clinical practice. Edema and fibrinous exudation can occur in the joint cavity of the cricoarytenoid joint after injury, and in the case of delayed treatment, they may lead to ankylosis of the cricoarytenoid joint with permanent voice damage and compromised airway protection [14]. Sataloff et al. [15] stated that performing closed reduction surgery within 10 weeks of dislocation can still achieve stable and good results, and even though late closed reduction surgery cannot restore joint movement, it may improve the voice quality of the patient. Lou et al. [16] investigated 34 patients with arytenoid dislocation and reported that performing closed reduction surgery within 2–4 weeks post-injury achieved satisfactory therapeutic effects and reduced the number of reduction attempts. Similarly, Lee et al. [17] suggest performing closed reduction surgery within 21 days of the injury to achieve good outcomes. However, Wu et al. [9] reported that performing closed reduction surgery within 24–48 h post-dislocation yields the best outcomes, and the earlier the treatment, the better the outcome. The criteria for successful reduction include restoration of vocal cord activity on the affected side, complete closure of both vocal cords and notable improvement in the voice quality. In the case reduction is ineffective, corticosteroids can be administered in small doses depending on the extent of joint mucosal swelling. The next closed reduction surgery should be attempted within 2–7 days after most of the swelling subsides. Generally, two–three closed reduction surgeries are required to achieve better outcomes for shorter illness durations. For patients with longer illness durations, closed reduction surgery may aid in voice recovery [9,15,18]. In the presented case, the patient achieved a favorable prognosis after two sessions of closed reduction surgeries.

Open surgical procedures are typically recommended for patients with dislocation lasting over 10 weeks or with poor outcomes after multiple closed reduction attempts, such as vocal fold injection, thyroid cartilage shaping, or arytenoid joint open reduction [19,20]. For patients who cannot tolerate surgery or have poor reduction outcomes, methods such as voice therapy, adjunctive anti-inflammatory medication, and botulinum toxin injections are employed. Rontal et al. [21] reported that intramuscular injection of the botulinum toxin into the larynx can relax relevant muscles, alleviating arytenoid dislocation. Furthermore, Tan et al. [22] reported a case of arytenoid dislocation where the voice of the patient returned to normal after 2 weeks solely through voice therapy.

This case report highlights LMA use as the potential cause for arytenoid dislocation in the clinical setting. Similar incidences may be prevented by strictly adhering to the indications and contraindications of LMA, selecting the appropriate size and type of LMA, checking the seal of the LMA before insertion, adequately lubricating the tips and sides to prevent tissue damage, using gentle insertion techniques while avoiding forceful maneuvers, ensuring that the inflation volume of the cuff does not exceed the maximum recommended volume, maintaining appropriate depth of anesthesia and muscle relaxation to increase the success rate of LMA insertion, and assessing the anatomical position and ventilation function of the LMA after insertion.

Recently, the development of visual LMA has rapidly progressed, offering the advantage of prompt adjustment of the LMA position and guiding endotracheal intubation. Notably, regardless of the model or brand, blind insertion of LMA can fail to achieve the ideal anatomical position in 50 %–80 % of cases [23,24]. However, employing visual LMA can achieve a 94 % rate of alignment between the mask opening and the glottis [25], potentially reducing the incidence of arytenoid dislocation.

## 4 Conclusion

In conclusion, arytenoid dislocation can considerably affect the quality of life of the patient. Postoperative vigilance is necessary for patients exhibiting symptoms such as persistent hoarseness and coughing while drinking or swallowing, followed by prompt diagnosis and early treatment, is necessary. The treatment should be initiated as soon as arytenoid dislocation is diagnosed to achieve optimal outcomes. Throughout the diagnostic and therapeutic process, patients and their families must be treated sincerely, and friendly doctor–patient communication occurs, ensuring their understanding and cooperation, along with paying attention to humane care and avoiding conflicts between doctors and patients.

## Consent

Written informed consent was obtained from the patient for publi-cation of this case report and accompanying images. A copy of the written consent is available for review by the Editor-in-Chief of this journal on request.

## Ethical approval

Ethical clearance is not required for this case report, according to our institution's research ethics committee.

## Funding

No funding.

## Author contribution

Lingxi Xing and Yuyan Ding contributed to writing and revising the manuscript. Lianbing Gu and Rong Gao contributed to the performing of anesthesia. Yihu Zhou contributed to the collection of data. Lixiang Yu contributed to revising the manuscript. All authors read and approved the final manuscript.

## Guarantor

Lianbing Gu.

## Research registration number

1. Name of the registry: The Affiliated Cancer Hospital of Nanjing Medical University & Jiangsu Cancer Hospital.
2. Unique identifying number or registration ID: None.
3. Hyperlink to your specific registration (must be publicly accessible and will be checked): None.

## Conflict of interest statement

All author declare that they have no conflicts of interest.
